# Voluntary Alcohol Intake following Blast Exposure in a Rat Model of Mild Traumatic Brain Injury

**DOI:** 10.1371/journal.pone.0125130

**Published:** 2015-04-24

**Authors:** Yi Wei Lim, Nathan P. Meyer, Alok S. Shah, Matthew D. Budde, Brian D. Stemper, Christopher M. Olsen

**Affiliations:** 1 Neuroscience Research Center, Medical College of Wisconsin, Milwaukee, Wisconsin, United States of America; 2 Department of Pharmacology & Toxicology, Medical College of Wisconsin, Milwaukee, Wisconsin, United States of America; 3 Department of Neurosurgery, Medical College of Wisconsin, Milwaukee, Wisconsin, United States of America; 4 Clement J. Zablocki Veterans Affairs Medical Center, Milwaukee, WI, United States of America; The Scripps Research Institute, UNITED STATES

## Abstract

Alcoholism is a frequent comorbidity following mild traumatic brain injury (mTBI), even in patients without a previous history of alcohol dependence. Despite this correlational relationship, the extent to which the neurological effects of mTBI contribute to the development of alcoholism is unknown. In this study, we used a rodent blast exposure model to investigate the relationship between mTBI and voluntary alcohol drinking in alcohol naïve rats. We have previously demonstrated in Sprague Dawley rats that blast exposure leads to microstructural abnormalities in the medial prefrontal cortex (mPFC) and other brain regions that progress from four to thirty days. The mPFC is a brain region implicated in alcoholism and drug addiction, although the impact of mTBI on drug reward and addiction using controlled models remains largely unexplored. Alcohol naïve Sprague Dawley rats were subjected to a blast model of mTBI (or sham conditions) and then tested in several common measures of voluntary alcohol intake. In a seven-week intermittent two-bottle choice alcohol drinking test, sham and blast exposed rats had comparable levels of alcohol intake. In a short access test session at the conclusion of the two-bottle test, blast rats fell into a bimodal distribution, and among high intake rats, blast treated animals had significantly elevated intake compared to shams. We found no effect of blast when rats were tested for an alcohol deprivation effect or compulsive drinking in a quinine adulteration test. Throughout the experiment, alcohol drinking was modest in both groups, consistent with other studies using Sprague Dawley rats. In conclusion, blast exposure had a minimal impact on overall alcohol intake in Sprague Dawley rats, although intake was increased in a subpopulation of blast animals in a short access session following intermittent access exposure.

## Introduction

Co-morbidity between traumatic brain injury (TBI) and substance use disorder (SUD), has been demonstrated in numerous epidemiological studies [1–4]. For example, in a prospective study of over 900 patients, TBI sufferers without psychiatric illness or SUD in the year prior to injury had an odds ratio of 4.5 of developing an SUD within 1 year of injury compared to those without TBI [5]. In the same study, rates of SUD prevalence rose from 7.3% pre-TBI to 14% 1 year post-injury, while rates of SUD in matched uninjured controls were 1.7% and 1.6% in the same respective time period [5]. Additionally, a study of subjects with no history of mental disease or SUD prior to TBI found that 18% were diagnosed with a SUD following injury [6]. Studies specifically investigating alcohol (ethanol) use following TBI have found no clear relationship between injury and drinking, although there is evidence that alcohol use declines in some patients and escalates in others. One study of self-reported alcohol use before and after TBI indicated that among patients that were moderate-heavy drinkers pre-injury, less than one third of them remained in this category, and approximately half of them had become abstinent [1]. Similar reductions in alcohol intake have been reported in patients surveyed one year following treatment [7]. On the contrary, one study published in 1994 found that 32% of TBI sufferers were alcohol dependent two years after injury [8], compared to a 4.4% prevalence of alcohol dependence among adults in the United States during a similar timeframe [9]. Other studies also suggest a specific increase in alcohol consumption following TBI. For example, of patients that reported little or no alcohol use pre-injury, approximately 10% had increased alcohol intake [1]. Another study looking at alcohol use patterns at least two years following injury noted a significant increase in alcohol consumption [2]. Taken together, these data indicate that there is no clear trajectory of alcohol use following TBI, however, they do suggest that there may be a bimodal distribution of alcohol intake amongst TBI patients following injury, where most patients are either abstinent or have moderate to high levels of drinking [10].

Additional factors complicate interpretation of clinical studies. Pre-injury substance use is often not reported or is measured using subjective scales [11]. Another complicating factor is that it is unknown whether post-injury alcohol use is due to neurological effects of TBI, is attributable to coping with pain or disability that may be associated with the injury, or if there is an interaction of factors [3]. For example, increased alcohol use has been strongly linked to post-traumatic stress disorder (PTSD), even in the absence of brain injury [12]. Although it is difficult to resolve these factors in clinical studies, there is evidence that both neurological and psychological factors can contribute to post-injury alcohol use. In a study of over 7000 military personnel with combat experience, odds ratios for frequent binge drinking exposures were elevated in TBI sufferers compared to those that experienced either no injury or a non-TBI injury [13] (although see [14]). Additionally, injury mechanism is typically not accounted for in clinical studies, with preclinical data demonstrating conflicting changes in rodent behavioral phenotypes between primary blast and the more common head impact/rotational acceleration mechanism. Those studies demonstrated that rats with brain injury resulting from shockwave exposure (i.e., primary blast) had elevated levels of post-injury anxiety-like behavior [15], while rats exposed to rotational acceleration TBI demonstrated a phenotype more consistent with behavioral disinhibition [16]. Finally, data from mild TBI (mTBI) is often combined with that from moderate and severe TBI, despite evidence of an inverse relationship between TBI severity and future alcohol use [17]. Mild TBI accounts for 75% of all traumatic brain injuries and there is evidence that patterns of alcohol intake may distinctly increase following mTBI [18].

As an initial step to address the effects of mTBI on subsequent alcohol use, we used a previously validated rodent blast TBI (bTBI) model and measured voluntary alcohol intake afterwards. Alcohol naïve rats were used to exclude interaction between any neuroadaptations and/or neurotoxicity associated with previous alcohol exposure and the blast injury. We specifically chose the Sprague Dawley rat strain because they display low alcohol intake under basal conditions [19,20] and neurotrauma associated with bTBI endures for at least 30 days in this strain [15]. Importantly, blast damage is particularly evident in the medial prefrontal cortex (mPFC) [15], a brain region highly implicated in drug and alcohol addiction [21–23], and commonly affected in human mTBI [24–27]. The overall aim of this study was to determine if bTBI would affect voluntary alcohol intake in alcohol naïve rats. mPFC dysfunction has been proposed to impair control over drug intake [21], possibly due to impaired processing of conflicts related to drinking behavior [28,29]. This theory has been supported by preclinical findings of mPFC neuroadaptations being associated with “aversion resistant” alcohol consumption [30,31]. Furthermore, chronic alcohol exposure was shown to induce mPFC dysfunction [32], which could exacerbate future drinking. We hypothesized that alcohol intake, especially during quinine adulteration tests (a measure of “aversion resistant” consumption), would be elevated in blast exposed rats.

## Materials and Methods

### Animals

A total of 52 male Sprague Dawley rats (Harlan, Madison, WI) were used in this study: 27 were used in the blast injury assessment experiment using the Composite Neuroscore, and 24 were used in the alcohol drinking study. Rats were approximately 8 weeks of age upon arrival to the Zablocki Veterans Affairs Medical Center. Rats were housed two to a cage until blast or sham treatment, at which time they were singly housed for the rest of the study. The length of the injury assessment study from the time of blast or sham treatment was 4 days. The length of the alcohol drinking study from the time of blast or sham treatment was 23–26 weeks. Food and water was provided *ad libitum* throughout the entire experiment. The animals were allowed to acclimate to the reverse light cycle (lights off 1200–2400) and housing conditions for at least 10 days prior to experimentation, and this light cycle was maintained at both facilities. Each rat in the alcohol drinking study went through all of the procedures in the order described (with the exception of the additional quinine adulteration test, which only a subset of rats were tested in). All experiments were approved by the Institutional Animal Care and Use committee at the Medical College of Wisconsin and were performed within the guidelines of the Guide for the Care and Use of Laboratory Animals (8^th^ Edition).

### Blast Exposure

Rats were exposed to either blast overpressure (450 kPa, 80 kPa*ms) or sham conditions as described [15,33] at the Zablocki Veterans Affairs Medical Center. Briefly, rats were anesthetized with Isoflurane (4%), then placed into a nosecone for continuous delivery of 1.5% Isoflurane. A metal cylinder was placed around the body to shield the torso from blast and the head was restrained to prevent injury due to head rotational acceleration. The animal was then placed 17 cm from the end of a custom shock tube (3.6 cm inner diameter, 3.0 m driven section, and 0.3 m driver section with mylar membrane between the driver and driven sections). To produce blast overpressure, the driver section was pressurized with helium until the membrane ruptured. Sham rats received the same treatment including anesthesia and placement in the holder, but were not exposed to shockwave overpressure. After blast, rats were observed until return of the righting reflex, then returned to the homecage and observed periodically for 6 hours. Rats in the alcohol drinking study were transferred to animal facilities at the Medical College of Wisconsin (~5 miles away) at least four days following exposure. Rats weighed 330–400g upon arrival and were individually housed in ventilated Plexiglas cages. Rats were then acclimated for >10 days prior to initiating the alcohol drinking studies.

### Injury Assessment

Time to recovery was assessed as the time taken to regain the righting reflex following termination of Isoflurane. This metric was assessed in both the blast injury assessment experiment and the alcohol drinking experiment. The Composite Neuroscore Motor Assessment was used to grade post-traumatic gross neurological dysfunction in the blast injury assessment experiment only. The task assessed six neurologically-related responses with a score between 0 (severely impaired) and 2 (normal). The composite score represents the sum of all tests, with a score of 12 representing no impairment and 0 representing the most severe impairment. The six tasks included: (1) left and (2) right forelimb flexion during tail suspension; (3) left and (4) right hind limb flexion with forelimbs remaining on a flat surface as hind limbs are lifted up and down by the tail; and ability to resist lateral pulsion to the (5) left and (6) right. Composite Neuroscore was quantified on post-injury day 4 by an observer blinded to the injury status of the rat using videos obtained during the test.

### Intermittent-access two-bottle choice

Rats were trained to consume 20% ethanol solution in hyperchlorinated water (standard drinking water) using the intermittent-access paradigm for three 24-hour sessions per week (Monday, Wednesday and Friday) as described [34–36]. In this two-bottle choice paradigm, they were given a choice to consume water or ethanol solution during the experiment. During abstinence days where ethanol solution was not present, two bottles filled with standard drinking water were presented instead. All fluids were presented in standard rodent drinking bottles with stainless-steel drinking spouts inserted through the cage wire bars 15 minutes after the lights went off (1215). The placements for both bottles were alternated every session to control for side preference. Both bottles were measured 24 hours after they were presented (including abstinence days) and measurements were taken to the closest 0.1g. During this and all other measures of drinking, ghost cages (i.e., no rat in the cage) were present on each row of the housing rack, and the amount of leak was calculated for each solution and subtracted from intake values. All the animals were weighed the same time when the bottles were weighed to calculate g/kg/24 hour of ethanol intake. This intermittent-access paradigm was carried out for seven weeks and blood was drawn from the lateral tail vein immediately after the last session of week seven (which was shortened to one hour) to analyze blood ethanol concentrations.

### Alcohol deprivation effect (ADE)

Rats next underwent three cycles of ADE testing. Each cycle was comprised of a two-week abstinence period (two bottles of water available) followed by a 24 hour two-bottle choice test with 20% ethanol. After the final ADE cycle, intermittent-access two-bottle choice with 20% ethanol and water was reestablished for one week before undergoing quinine adulteration and preference testing.

### Quinine adulteration/preference tests

Quinine adulteration tests were performed during the first session (Monday) of intermittent-access each week, similar to that described [37]. Rats were presented with 20% ethanol containing different doses of quinine. Testing took place over five weeks, where quinine adulteration of alcohol occurred on Mondays, and 20% ethanol two-bottle choice (without quinine adulteration) occurred on Wednesdays and Fridays. Rats were presented with two bottles of drinking water on other days of the week. Each rat was given each quinine dose of 0, 0.003, 0.01, 0.03, and 0.1g/L in the 20% ethanol solution using a Latin square design. Side placements for the quinine/alcohol-containing bottles were counterbalanced between animals. After this test, rats underwent a quinine preference test where each concentration of quinine was presented in a Latin square design for 48 hours using the two-bottle choice (drinking water vs. quinine + drinking water) paradigm. Bottle sides were switched every 24 hours. A subset of animals was then subjected to an additional quinine adulteration test under the same conditions described above, except the doses were 0, 0.03, 0.3 g/L in 20% ethanol and were presented in descending order across three weeks.

### Blood ethanol concentration assay

Immediately after the one hour intermittent-access two-bottle choice session (the final session in the seven week intermittent-access paradigm), whole blood was collected into vacutainer plastic serum tubes, allowed to clot at RT for 30–60 minutes, then centrifuged at 4° C for 10 minutes at 2560 rcf. Serum was separated and stored at -80°C until analysis. All sera were then analyzed using a colorimetric ethanol assay kit (Sigma Aldrich, cat# MAK076) and the blood ethanol concentration was determined based on the standard curve obtained using a spectrophotometer (Molecular Devices Spectramax M3).

### Analysis

Data were analyzed by two-tailed Student’s t-test, Mann-Whitney U (when data did not have a normal distribution) and ANOVA (repeated measures when appropriate) followed by between-group Holm-Sidak multiple comparisons using Prism 6.0. Significance was set at p = 0.05.

### Ethics Statement

This study was carried out in strict accordance with the recommendations in the Guide for the Care and Use of Laboratory Animals of the National Institutes of Health (8^th^ Edition). The protocol was approved by the Institutional Animal Care and Use Committee of the Medical College of Wisconsin (Permit Number: AUA 2816). Blast procedures were performed under Isoflurane anesthesia, and all efforts were made to minimize pain and/or suffering.

## Results

### Acute recovery from blast/sham procedure

One animal (sham) was removed from the study due to having a Isoflurane recovery time >5 standard deviations from the mean. In the blast injury assessment experiment, there was no significant difference in time to regain righting reflex between sham and bTBI rats (sham: 204.0 ± 25.3 sec, bTBI: 156.1 ± 17.7 sec, t(24) = 0.81, n.s.). Likewise, in the alcohol drinking experiment, there was no significant difference in time to recover from Isoflurane induced loss of consciousness between sham or bTBI rats (sham: 127.9 ± 12.6 sec, bTBI: 136.2 ± 9.8 sec, t(22) = 0.52, n.s.).

### Neuromotor function following blast/sham procedure

Four days following sham/bTBI, rats in the blast injury assessment experiment were tested for neuromotor function using the Composite Neuroscore. There was no significant difference in this measure between sham and bTBI rats (sham: 10.7 ± 0.63, bTBI: 10.03 ± 0.54, t(24) = 0.81, n.s.).

### Intermittent-access two-bottle choice

**Fig 1:**
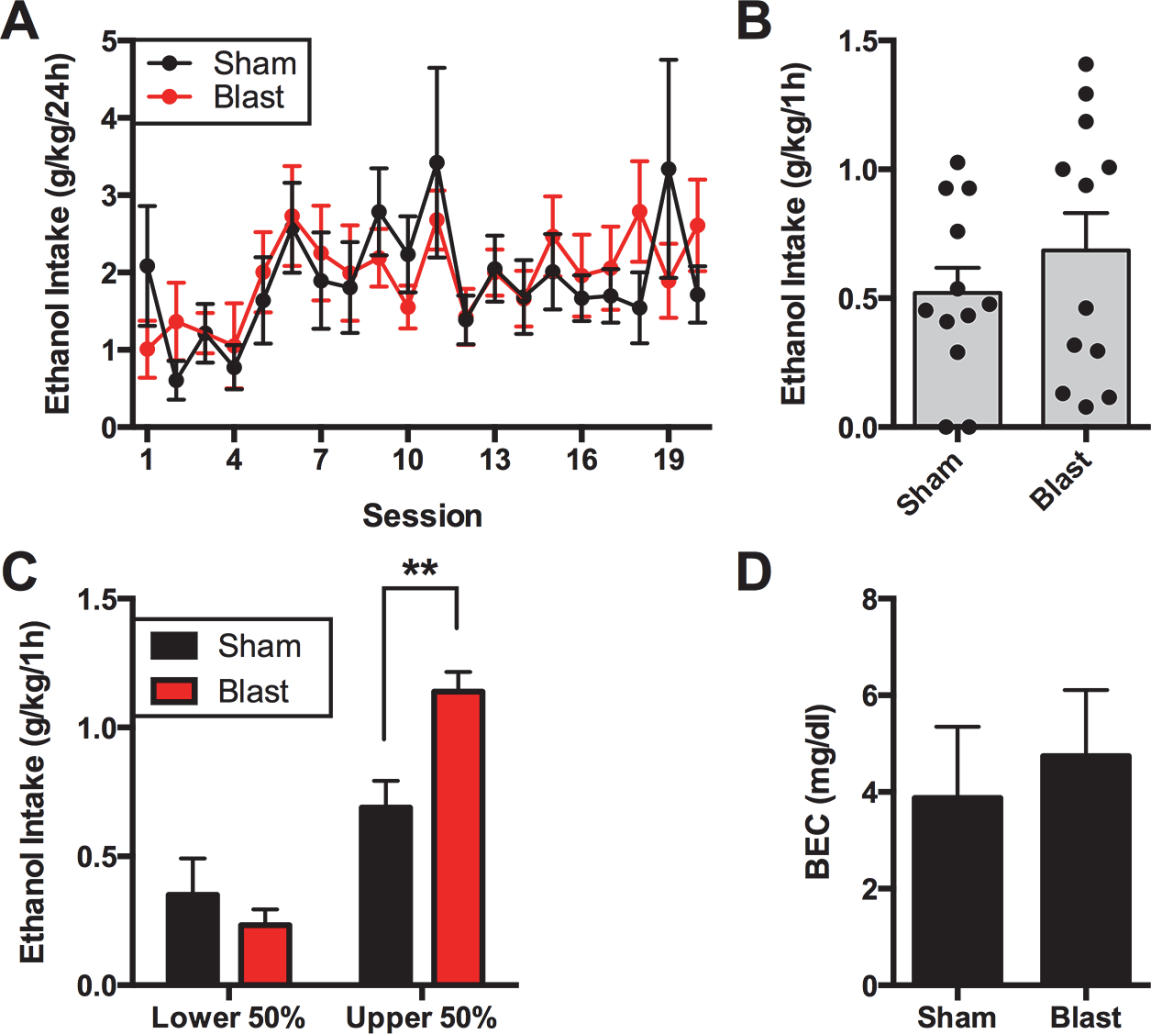
Ethanol intake during intermittent access 20% ethanol two-bottle choice. A) Intake during seven weeks (20 sessions) of two-bottle choice. Tick marks denote the first session of each week. B) Ethanol intake during a 1 hour two bottle choice session (session 21). Blast animals fell into a bimodal distribution, prompting a median split of the data. C) Ethanol intake in lower and upper intake subgroups of sham and blast animals based on median split. **p<0.01. D) Blood ethanol concentration (BEC) in high intake subgroups (n = 6/group). Tail vein blood was collected from rats immediately following a one-hour drinking session and assayed for BEC. Bars represent mean ± SEM, individual data points shown in B.

After blast exposure (or sham treatment) and recovery (12–14 days), rats underwent an intermittent two-bottle choice test using 20% ethanol [34,35] to determine if blast exposure would affect subsequent alcohol drinking. In the first 20 sessions, there was a main effect of session (F(19, 418) = 2.476, p<0.001), but not blast treatment (F(1,22) = 0.01, n.s.), nor was there an interaction between factors (F(19,418) = 0.90, n.s.) (Fig 1A). An additional session was conducted for one hour to measure alcohol intake and corresponding blood ethanol concentrations (BECs) during this time. There was no difference in ethanol intake between groups (U = 57, n.s.); however, the blast group had a bimodal distribution of ethanol intake (Fig 1B). We reanalyzed ethanol intake following a median split of each group to determine if there were differences within the upper and lower intake subpopulations (Fig 1C). There was a significant main effect of the median split (F(1,20) = 38.44, p<0.0001), but not blast treatment (F(1,20) = 2.74, n.s.). However, there was an interaction between the population split and blast treatment (F(1,20) = 8.0, p<0.05). *Post hoc* analysis demonstrated that amongst the upper intake animals, blast exposed rats had significantly higher intake (p<0.01). Despite this difference in intake, there was no significant difference in BECs between treatment groups (U = 15, n.s.) (Fig 1D).

### Alcohol deprivation effect

**Fig 2:**
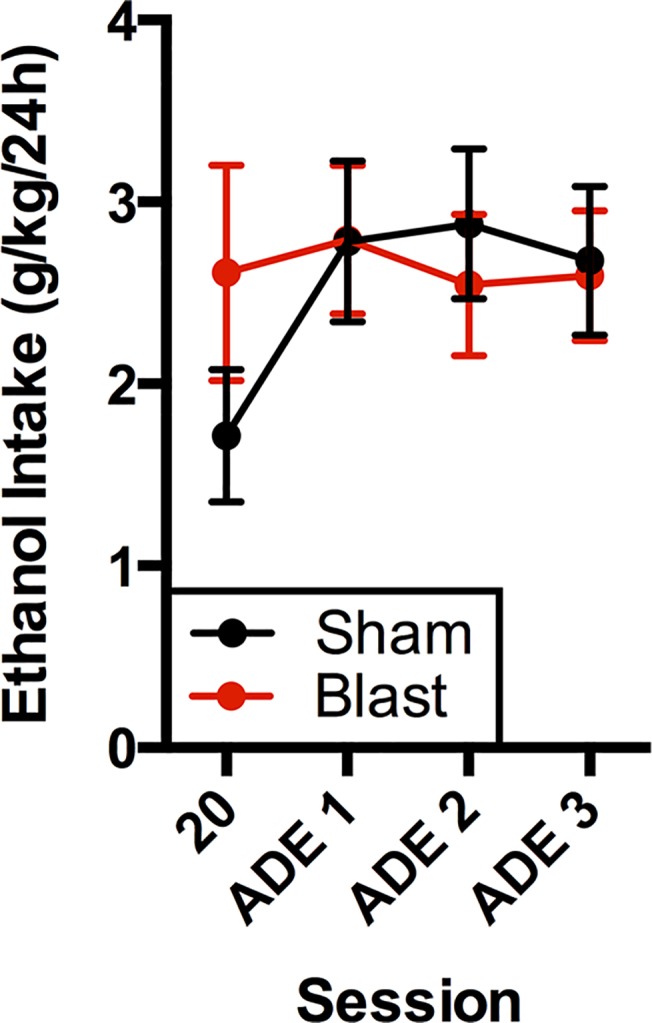
Ethanol intake after deprivation periods. Rats were tested in two-bottle choice (20% ethanol/water) sessions that started two weeks after intermittent access two-bottle choice sessions. Each session was 24 hours in length and had a two-week alcohol deprivation period prior to it. Session 20 (the final 24 hour session during the intermittent access phase) and three ADE sessions are shown for comparison. Bars represent mean ± SEM.

Beginning two weeks after intermittent-access two-bottle choice alcohol drinking, rats were tested under conditions that have been reported to induce an increase in alcohol drinking, known as an alcohol deprivation effect [38,39]. For each of the three tests, rats were given 24-hour access to alcohol under the same two-bottle choice conditions described above with two weeks of abstinence in between each session (Fig 2). There were no significant differences between treatment groups under ADE conditions (F(1,22) = 0.07, n.s.), nor was there an effect of session number (F(3,66) = 1.53, n.s.) or interaction (F(3,66) = 1.38, n.s.). Within-subjects planned comparisons verified an ADE in sham treated animals (intake increased in each ADE session relative to session 20, p<0.05), but not blast treated animals.

### Quinine adulteration/preference tests

**Fig 3:**
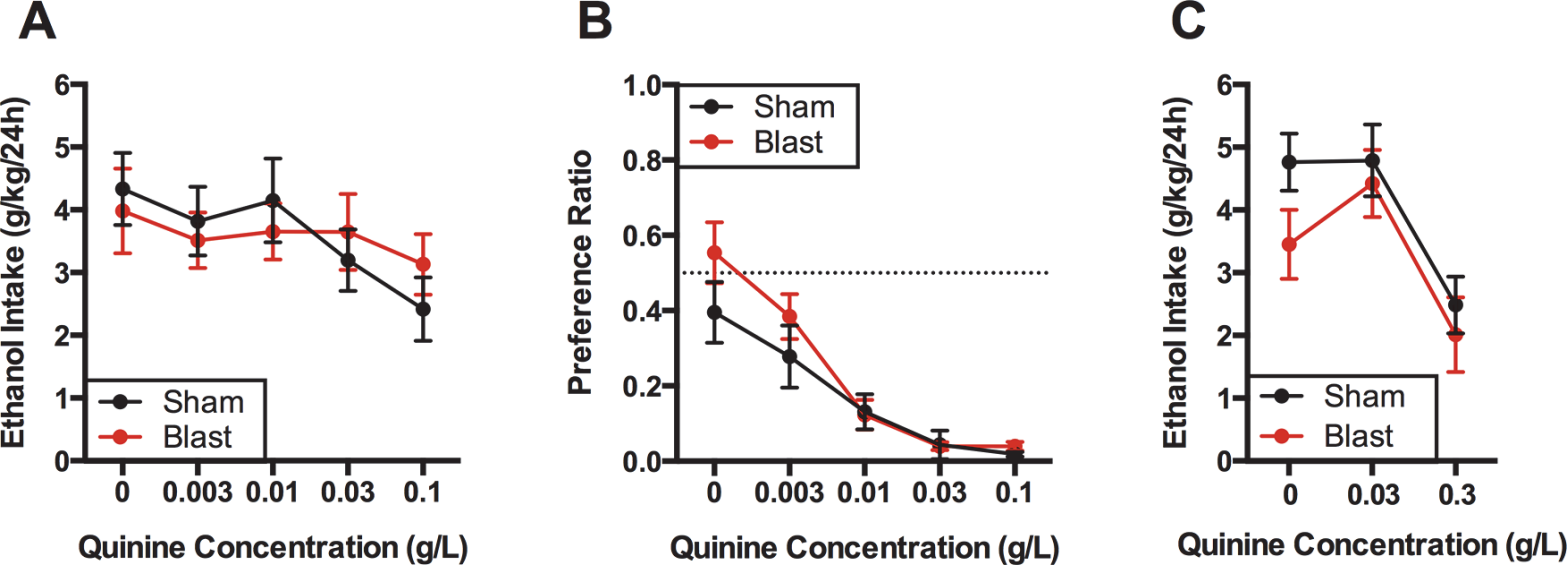
Quinine adulteration and taste preference tests. A) Quinine was added to ethanol in weekly tests of quinine adulteration in a two-bottle choice paradigm (water vs. 20% ethanol+quinine). B) Quinine preference was determined in a two-bottle choice paradigm (water vs. water+quinine). C) An additional quinine adulteration test was performed in a subset of rats with a higher maximum quinine concentration. N = 12/group in A, B. N = 6-9/group in C.

After ADE testing, rats underwent a test of quinine adulteration of 20% alcohol as a measure of compulsive alcohol drinking [30,37,40] (Fig 3A). As expected, quinine reduced ethanol intake (F(4,88) = 3.4, p<0.05), but there was no effect of blast treatment (F(1,22) = 0.0, n.s.) or interaction of quinine concentration and treatment (F(4,88) = 0.89, n.s.). To determine if there was any difference in the ability of quinine to reduce taste preference amongst groups, rats were tested for preference of each concentration of quinine used vs. that of water (Fig 3B). Quinine sharply shifted preference in a concentration-dependent manner (F(4,88) = 28.9, p<0.0001), and there was no effect of treatment (F(1,22) = 1.9, n.s.) or interaction (F(4,88) = 1.0, n.s.) on this preference. Because quinine had a modest effect on suppressing alcohol intake in the initial test, we performed an additional quinine adulteration test with an extended concentration range in a subset of rats (Fig 3C). In this test, rats were exposed to three concentrations of quinine in 20% ethanol. This again yielded a significant effect of quinine concentration (F(2,26) = 15.8, p<0.0001), although there was no effect of treatment (F(1,13) = 1.6, n.s.) or interaction (F(2,26) = 0.68, n.s.).

### Re-analysis of upper intake subjects

Considering that bTBI only had a significant effect on the upper intake rats during short access two bottle choice, we re-examined the data using only these subjects. There were no significant main effects of blast or interaction effects in any of the following measures: Intermittent-access two-bottle choice (treatment (F(1,10) = 0.35, n.s., interaction (F(19,190) = 0.96, n.s.)), alcohol deprivation effect (treatment (F(1,10) = 0.12, n.s., interaction (F(2,20) = 0.18, n.s.), quinine adulteration (treatment (F(1,10) = 0.50, n.s.), interaction (F(4,40) = 0.98, n.s.), or quinine preference (treatment (F(1,10) = 0.25, n.s.), interaction (F(4,40) = 1.23, n.s.).

## Discussion

In this study, we used a low alcohol drinking outbred rat strain, Sprague Dawley, to assess the impact of mTBI on alcohol drinking. Injury assessment by recovery from sham/bTBI and Composite Neuroscore assessed four days later indicated no significant differences between sham and bTBI rats, consistent with a mild injury. In regards to alcohol drinking, we found that during a seven-week intermittent access phase, there was no effect of blast exposure on intake of 20% alcohol during the 24-hour drinking sessions. On the final session, access was terminated one hour following alcohol availability to investigate alcohol intake during this initial period of access. We found that among blast treated rats, intake levels during the one-hour session fit a bimodal distribution. This bimodal pattern of intake has been previously reported with Sprague Dawley rats [41], although more homogenous variability has also been observed in this outbred strain [34]. We performed a median split of the data and compared the effect of blast exposure in upper and lower intake rats. Alcohol intake was significantly elevated among upper intake rats exposed to blast compared to sham conditions, while there was no difference between lower intake rats. Despite the differences observed in alcohol intake, BECs were not different between treatment groups during the one-hour session. The reason for this discrepancy is not clear, although it may be based on the timing of our measurements. Many studies measuring BECs following alcohol intake in the rat do so after 30 minutes of access (e.g., [34,35,42]). During the two-bottle choice phase in our study, it was noted that most rats were not drinking in the initial 30 min of access, and thus we chose to use a one-hour session for determining BEC following voluntary intake. This longer time could have increased the variability in timing of intake and subsequent absorption and metabolism of ethanol in such a way that BECs did not consistently reflect intake. Although this may indicate that the timing of drinking was different between sham and bTBI rats, it should be noted that the correlation between ethanol intake and blood ethanol concentrations is not always robust with the relatively low intake levels observed in the current study (e.g., [34,43]). We also measured alcohol drinking following periods of abstinence to measure potential differences in the “alcohol deprivation effect” [38,39]. Although we confirmed the presence of an ADE in sham animals, we found no effect of blast treatment on alcohol drinking during any of these sessions. Finally, to measure compulsive alcohol drinking, alcohol was adulterated with different concentrations of quinine. We found no effect of blast treatment on the reduction of alcohol intake by quinine, nor did we observe any difference in quinine aversion in the preference test. Thus, across several measures of alcohol intake, a significant effect of blast was only observed when the upper 50% of rats in each group were compared in a short access two bottle choice session. To determine if there was a significant effect of blast in these animals in other measures of intake, other measures were re-analyzed using only the upper intake rats. There were no significant effects observed in this re-analysis, suggesting that this divergence in the blast population was specific to short access alcohol drinking.

Preclinical studies to dissociate factors contributing to alcohol use following TBI have recently emerged. Thus far, these studies suggest complex interactions between neurological damage, alcohol exposure, and psychological resilience. For example, in rats with previous exposure to alcohol, TBI increased alcohol self-administration on subsequent sessions [44]. Alcohol exposure following TBI was also associated with impaired neurological recovery and elevated levels of the neuroinflammatory markers GFAP, ED-1, and HMGB1 [45]. In addition to these factors, increased stress reactivity (a preclinical model of PTSD) was also identified as a factor associated with higher alcohol self-administration [30]. There is also evidence of alcohol exacerbating PTSD-associated phenotypes, as chronic alcohol exposure was found to impair extinction of a fear memory and decrease activity of mPFC neurons during extinction training [32]. These studies suggest that even in rodent models, alcohol intake is likely modulated by both neurological and psychogenic factors. Future research is needed to delineate the distinct and interactive roles of brain injury and stress reactivity on patterns of substance use.

The bTBI model used induces significant neurotrauma in several brain regions, including the mPFC [15]. The mPFC has been highly implicated in drug and alcohol addiction [21–23]. Alcoholism has been associated with loss of grey matter in the mPFC [46,47] and impairment in mPFC-dependent decision-making tasks [22]. Furthermore, increased atrophy within the mPFC was associated with increased relapse rates in alcohol-dependent individuals [47]. In preclinical models, impairment of mPFC has also been associated with aversion-resistant alcohol intake [30,31]. Considering the evidence for mPFC involvement in the regulation of alcohol intake, it is notable that there was no significant effect of blast exposure in the majority of our measurements.

There are several technical considerations in the present study that likely contributed to these outcomes. First, bTBI was administered prior to any exposure to alcohol. It is well known that establishing voluntary alcohol intake in rats not selectively bred for alcohol preference or intake occurs slowly over several weeks in the absence of forced exposure or sucrose fading [48–50], and effects of the bTBI may have waned during this time. This approach was chosen in an effort to isolate the neurobehavioral effects of blast from any potential effects associated with prior alcohol exposure or interaction of blast and prior alcohol exposure. In clinical settings, however, the majority of TBI victims have a history of alcohol use, and many are intoxicated at the time of injury [1,2,11,51]. There is also evidence that TBI victims with higher alcohol intake prior to injury are more susceptible to increased drinking after injury [7], and a recent preclinical study found that TBI-associated increases in alcohol intake were positively correlated with pre-injury levels of intake [44]. Considering the lack of significant effect of bTBI on the group as a whole in the present study, these data are consistent with the aforementioned studies indicating that the effects of TBI may be most robust in individuals with high intake prior to injury. The reason for the increase in short access ethanol intake only in a subset of bTBI rats is unclear, but may reflect individual differences in injury extent, susceptibility, or other factors. Future studies are needed to investigate effects of prior and/or concurrent alcohol exposure on alcohol intake following bTBI. A second consideration is the strain of rat used: Sprague Dawley rats have low to moderate voluntary alcohol intake [19,20]. We chose this strain for two primary reasons. First, this is the rat strain used in our previous characterization of bTBI [15]. Second, we wanted to determine if blast exposure could increase ethanol intake in rats that are resistant to high voluntary alcohol intake under normal circumstances. Although we have found that this blast model leads to microstructural changes in the mPFC that last for at least 30 days [15], the majority of our testing was done at later timepoints (30 days post-injury corresponds to the first half of the intermittent access two-bottle choice phase of this experiment). There were no measures of microstructal damage made in the current study, and thus it is unclear whether variations in blast injury were associated with intake in any measures or whether injury persisted for the duration of our testing.

The biomechanics of the present experimental model were specifically designed to produce mild traumatic brain injury in rats through interaction of the shockwave overpressure with brain tissues [15]. Induction of mild blast TBI has been the focus of a number of prior experimental models incorporating primarily shock tube injury models. Unfortunately, direct comparison of blast TBI models is somewhat complicated by differing experimental conditions attributable to different experimental focus. For example, a number of prior studies were focused on providing whole-body exposure to shockwave overpressure [52–54], whereas other studies provided body shielding to limit brain injury attributable to pulmonary effects such as vasospasm [55,56]. Other injury models have produced considerable head accelerations during blast exposure [57,58], that exceeded thresholds for rotationally-induced TBI in the rat [16], and resulted in theories regarding polytraumatic brain injury due to combined effects of shockwave overpressure and head rotational acceleration during blast. The current focus was to produce mild blast TBI using the shockwave mechanism and limiting confounding effects due to pulmonary disruption and head rotational acceleration. The current data and previous characterization of this blast model using the same peak overpressure and shockwave impulse (450 kPa, 80 kPa*ms) indicate that injury is consistent with that of a mild TBI (e.g., no significant effect of bTBI on recovery time or Composite Neuroscore, no hemorrhage; also see discussion below) [15].

Ignoring differences in experimental protocols highlighted above, shockwave exposures can be compared between the present study and prior investigations. Peak shockwave overpressure is the most common metric reported for studies incorporating shock tube models. Peak overpressure from the current study (450 kPa) was considerably higher than prior work incorporating the Walter Reed Army Institute of Research (WRAIR) model (116–147 kPa) [52,56] or other experimental models (69–236 kPa) [59,60], although Svetlov and colleagues incorporated overpressures more in line with the current study (358 kPa) [58]. However, peak overpressure is not the best correlate for injury onset and severity, and an assessment of overpressure duration must be considered [61]. Accordingly, shockwave impulse, computed as the area beneath the positive portion of the overpressure versus time pulse, is likely a better indicator of the severity of blast shockwave exposure. Assuming shockwave profiles fit the Friedlander profile, shockwave impulse can be computed based on peak overpressure and positive duration characteristics. Shorter durations (0.5 ms) incorporated using the current model [33] result in shockwave impulse values (80 kPa*ms) that are more in line with prior literature. Longer durations produced by the WRAIR model (3–4 ms) [53] and other groups (1–2 ms) [60], combined with lower peak overpressures (116–236 kPa) result in shockwave impulse values between 50 and 216 kPa*ms. Other models incorporating very long durations (10–18 ms) [54,58] have considerably greater impulse values (640–1,000 kPa*ms) that presumably produce more significant injuries. Therefore, although the peak overpressure metric is somewhat higher in the current model, a more accurate assessment of the severity of shockwave exposure places the current model in line and somewhat on the lower end of a majority of prior work in this area.

Accordingly, the injury severity produced using the current experimental model is considered to be mild, based on exposure severity characteristics (described above) as well as behavioral and pathological outcomes. Behavioral changes resulting from the current TBI model were previously shown to include modest (albeit statistically significant) cognitive deficits and increased anxiety-like behavior assessed using the Morris Water Maze and Elevated Plus Maze [15]. These changes were evident at acute (< 7 days) and subacute (30 days) time points, but resolved by 60 days post injury. Behavioral deficits previously identified in the current model were generally consistent with other investigations incorporating cognitive [54,56] and anxiety [62] phenotype assessments in rodents following blast exposure. Histological examination following blast exposure using the current model revealed increased astrocyte hypertrophy, neuropathology, and apoptotic cells following 450 kPa exposures that were consistent with other models [63,64], although intracranial hematoma and brain swelling were not apparent. Models incorporating more severe injury models (358 kPa, 1,317 kPa*ms) reported massive intracranial hematoma and brain swelling [58]. Considerably less severe exposures (147 kPa, 160–215 kPa*ms) also produced hemorrhage, extensive necrosis, and extended apnea [56] that did not occur using the current model and were not evident for lower severity exposures in that study.

We found that among rats in the upper 50% of intake during a one-hour access session following 20 sessions of intermittent access, blast rats had higher alcohol intake than shams. Studies using Sprague Dawley rats have previously described high and low intake individuals, and mechanistic differences associated with intake have been described between high and low intake rats in this strain. For example, the orexin/hypocretin-1 receptor antagonist SB-334867 was found to reduce ethanol drinking in high, but not low intake rats [33]. Differences between high and low intake animals have also been noted within the prefrontal cortex. Morganstern, et al. [34] found that high intake rats had elevated opioid signaling relative to low intake rats, as evidenced by increased enkephalin and mu receptors in the mPFC. Considering the genetic heterogeneity present in the (outbred) Sprague Dawley strain, it is possible that TBI induces changes in neural function that interact with genetic differences and/or neuroadaptations induced by alcohol to specifically affect high intake rats. It is tempting to posit that a similar phenomenon is responsible for the divergence in human alcohol drinking following TBI, although this is highly speculative.

To date, we are only aware of two other published studies using an animal model of TBI to assess effects of brain injury on voluntary alcohol intake. In a study using an open head injury (lateral fluid percussion, LFP) model in rats with prior alcohol drinking, it was found that TBI increased alcohol intake, and this increase positively correlated with prior intake levels [44]. Another study used a non-contusion impact (NCI) model in the mouse [65], and found increased sensitivity to the sedative effects of an acute alcohol treatment and decreased alcohol intake in a drinking in the dark model. Although these studies appear to have conflicting findings, several important differences exist between the TBI models and other aspects of the experimental designs employed. The first difference is the degree of injury induced by the TBI model. The NCI model resulted in a >5 fold increase in time to recovery following anesthesia, compared to ~2 fold increase in the LFP model, and a non-significant difference (< 10%) in the present study [44,65]. Additionally, the blast model employed here does not produce skull fracture or localized bleeding [15], results that were reported in the NCI injury by Lowing et al. [65]. Another important difference is in the species and strain used. Our study used a rat strain that has low to moderate voluntary alcohol intake [19,20], while Wistar rats (more commonly used in voluntary ethanol drinking studies) were used in [44], and Lowing et al. [65] used C57BL/6 mice, which are characterized by high voluntary alcohol intake [66]. Compared to the others, our study investigated a longer timecourse and incorporated additional measures of drinking following abstinence and quinine adulteration. Nonetheless, these and future studies will be important in understanding the neurological and behavioral consequences of TBI on alcohol use and dependence.
